# Random mutagenesis-based screening of the interface of phyllogen, a bacterial phyllody-inducing effector, for interaction with plant MADS-box proteins

**DOI:** 10.3389/fpls.2023.1058059

**Published:** 2023-03-28

**Authors:** Yugo Kitazawa, Nozomu Iwabuchi, Kensaku Maejima, Oki Matsumoto, Masato Suzuki, Juri Matsuyama, Hiroaki Koinuma, Kenro Oshima, Shigetou Namba, Yasuyuki Yamaji

**Affiliations:** ^1^ Department of Agricultural and Environmental Biology, Graduate School of Agricultural and Life Sciences, The University of Tokyo, Tokyo, Japan; ^2^ Faculty of Bioscience and Applied Chemistry, Hosei University, Tokyo, Japan

**Keywords:** MADS-box transcription factors, phyllogen, protein-protein interaction (PPI), protein structure prediction (PSP), random mutagenesis

## Abstract

To understand protein function deeply, it is important to identify how it interacts physically with its target. Phyllogen is a phyllody-inducing effector that interacts with the K domain of plant MADS-box transcription factors (MTFs), which is followed by proteasome-mediated degradation of the MTF. Although several amino acid residues of phyllogen have been identified as being responsible for the interaction, the exact interface of the interaction has not been elucidated. In this study, we comprehensively explored interface residues based on random mutagenesis using error-prone PCR. Two novel residues, at which mutations enhanced the affinity of phyllogen to MTF, were identified. These residues, and all other known interaction-involved residues, are clustered together at the surface of the protein structure of phyllogen, indicating that they constitute the interface of the interaction. Moreover, *in silico* structural prediction of the protein complex using ColabFold suggested that phyllogen interacts with the K domain of MTF *via* the putative interface. Our study facilitates an understanding of the interaction mechanisms between phyllogen and MTF.

## Introduction

Proteins exert their functions through interactions with target molecules, and amino acid residues on the interaction interface play a regulatory role in interaction specificity and affinity. Thus, identifying the residues involved in the interaction, and their locations on the interaction interface, is important for understanding protein functions. Random mutagenesis, in combination with efficient selection methods, is a conventional but powerful and prevailing approach to map interaction-involved residues and identify the protein substructures involved in the interactions (Bertolaet et al., 2001; Vyncke et al., 2019; Rose et al., 2020). Error-prone polymerase chain reaction (PCR), in which mutations are inserted randomly into gene sequences during PCR under low-fidelity conditions, is the most common method for random mutagenesis (McCullum et al., 2010).

Phytoplasmas (‘*Candidatus* Phytoplasma’ spp.) are a group of plant pathogenic bacteria infecting more than 1,000 plant species (Marcone, 2014). Their infection induces various unique developmental abnormalities in plants (Namba, 2019). One of the most intriguing symptoms is phyllody (transformation of floral organs into leaf-like structures), which is induced by a phytoplasma-conserved effector family designated as phyllogen (phyllody-inducing gene family) (MacLean et al., 2011; Maejima et al., 2014). Phyllogen induces phyllody by targeting floral MADS-box transcription factors (MTFs), which are functionally classified into four classes (A, B, C, E) and form heterologous tetramers that specify the different floral organs (floral quartet model) (Honma and Goto, 2001; Theißen et al., 2016). Phyllogen interacts with and induces proteasome-mediated degradation of specific MTFs, including A- and E-class MTFs, but is not known to interact with B- and C-class MTFs (MacLean et al., 2014; Maejima et al., 2014; Maejima et al., 2015; Aurin et al., 2020). Phyllogen also interacts with RADIATION SENSITIVE23 (RAD23) (MacLean et al., 2014), which delivers ubiquitinated proteins to the proteasome (Dantuma et al., 2009). Our recent study indicated that phyllogen interacts with its target MTF, and then with RAD23 without ubiquitin, resulting in delivery to the proteasome and degradation of the MTF in a ubiquitin-independent manner (Kitazawa et al., 2022).

The A–E-class MTFs belong to type II MTF, which consist of a MADS (M) domain required for DNA-binding, followed by plant-specific intervening (I), keratin-like (K), and C-terminal (C) domains (Smaczniak et al., 2012). K domain is involved in the dimerization and tetramerization of MTFs (Yang and Jack, 2004; Lai et al., 2019). The crystal structure of the homotetramer of the K domain of SEPALLATA3 (SEP3; an E-class MTF of *Arabidopsis thaliana*) was determined, and two regions in the domain, which are respectively involved in self-dimerization and self-tetramerization, were identified (Puranik et al., 2014). Previous reports have identified that the K domain is recognized by phyllogen (MacLean et al., 2014; Liao et al., 2019). Recently, it was shown that phyllogen interacts with the self-tetramerization-involved region of SEP3 K domain, not the dimerization-involved region (Kitazawa et al., 2022). The interaction of phyllogen with SEP3 requires the five residues in the tetramerization-involved region of SEP3 K domain (methionine at position 150 and leucine at positions 154, 157, 164, and 171) (Kitazawa et al., 2022), which are highly conserved among E-class MTFs and responsible for its self-tetramerization (Puranik et al., 2014; Rümpler et al., 2018).

As for phyllogen, which consists of two alpha-helices connected by a loop (Iwabuchi et al., 2019; Liao et al., 2019), several residues involved in the interaction with MTF have been identified by site-directed mutagenesis analyses (Liao et al., 2019; Aurin et al., 2020; Iwabuchi et al., 2020). For example, Iwabuchi et al. (2020) demonstrated that two amino acid polymorphisms, at positions 30 and 64, respectively, affect the interaction affinity with MTF by swapping sequences of phyllody-inducing [lysine at 30 (K30) and asparagine at 64 (N64)] and non-phyllody-inducing [glutamine at 30 (Q30) and arginine at 64 (R64)] phyllogens. However, because these studies addressed a limited number of residues in an individual and arbitrary manner, the surface at which phyllogen interacts with MTF remains to be elucidated.

In this study, we comprehensively explored the residues of phyllogen at which mutations enhanced the affinity to MTF by using the error-prone PCR-based random mutagenesis approach. We also analyzed the positional relationships of the residues based on the structure of the phyllogen protein.

## Material and methods

### Plant materials and plasmid construction

*A. thaliana* (Col-0) was maintained in a growth chamber under 16 h light/8 h dark conditions at 22°C. *Nicotiana benthamiana* was grown under natural light conditions at 25°C. Information and abbreviation of phyllogens used in this study were listed in **Supplementary Table 1**. The primers and plasmids used in the study are listed in **Supplementary Tables 2** and **3**, respectively. Three phyllogens were cloned in the study: *PHYL_JHP_* from the ‘*Ca.* P. japonicum’ Japanese hydrangea phyllody (JHP) strain; *PHYL_RYD_* from the ‘*Ca.* P. oryzae’ rice yellow dwarf (RYD) strain; *PHYL_WBDL_* from the ‘*Ca.* P. aurantifolia’ witches’ broom of lime (WBDL) strain. *PHYL* genes were previously described as *PHYL1* but were renamed by Kitazawa et al. (2022). DNA fragments of the putative secreted regions of the genes were synthesized by Thermo Fisher Scientific (Waltham, MA, USA) with plant codon optimization (**Supplementary Data 1**). The optimized and the original sequences were compared in **Supplementary Figure 1**. The synthesized fragments were cloned into pENTA (Himeno et al., 2010), as described previously (Kitazawa et al., 2017). Other phyllogens used in this study were cloned in previous studies with plant codon optimization (Maejima et al., 2014; Kitazawa et al., 2017; Iwabuchi et al., 2019; Iwabuchi et al., 2020; **Supplementary Table 3**). Single amino acid mutants of phyllogen homologs were created by the GeneArt™ Site-Directed Mutagenesis System (Thermo Fisher Scientific) using appropriate primer pairs. Other plasmids for phyllogen expression were constructed as described below. In *Arabidopsis* MTF genes used in the study, *SEP3* (AT1G24260) was cloned previously (Maejima et al., 2014; Iwabuchi et al., 2019; Iwabuchi et al., 2020). *SHORT VEGETATIVE PHASE* (*SVP*; AT2G22540) was cloned in the study as described below.

### Error-prone PCR

For the construction of mutant libraries of the GAL4 binding domain (BD)-fused PHYL_OY_ (BD-PHYL_OY_), non-codon-optimized *PHYL_OY_* from the ‘*Ca.* P. asteris’ onion yellows (OY) strain (*PHYL_OY_org*; Maejima et al., 2014) was amplified by error-prone PCR using the Diversify PCR Random Mutagenesis Kit (Takara Bio, Shiga, Japan) and cloned into pGBKT7 (Takara Bio). PCR was repeated twice as described in the user manual (Condition 8), using the PhylF4 and PHYLorg_to_pGBKT7_R primers. The PCR conditions were 94°C for 30 s; 25 cycles at 94°C for 30 s, 50°C for 30 s, and 68°C for 1 min; and a final extension at 68°C for 5 min. For the preparation of mutant libraries of BD-fused PHYL_SY_, the plant codon-optimized *PHYL_SY_* of the ‘*Ca.* P. fragariae’ strawberry yellows (SY) strain (Iwabuchi et al., 2020) was also amplified by the same protocol using the SY_F and pGBK-fragariae-R primers. pGBKT7 was PCR-amplified by KOD One PCR Master Mix (Toyobo, Osaka, Japan) using the pGBKT7-F/-R primers.

The error-prone PCR amplicons were fused to the pGBKT7 fragment by recombinant PCR using KOD One PCR Master Mix. The first PCR was performed to fuse the DNA fragments without primers under the following conditions: 96°C for 3 min; 10 cycles of 96°C for 15 s, 55°C for 30 s, and 68°C for 40 s; and a final extension at 68°C for 4 min. Next, the fused fragment was amplified by step-down PCR with the appropriate primers (PHYLorg_to_pGBKT7_F and pGBKT7-R for PHYL_OY_ or pGBK-fragariae-F and pGBKT7-R for PHYL_SY_). The PCR conditions were as follows: 96°C for 3 min; five step-down steps, each comprising two cycles of denaturation at 98°C for 10 s, annealing from 68°C down to 56°C for 30 s (in 3°C steps) and elongation at 68°C for 40 s; 25 cycles of 96°C for 15 s, 55°C for 30 s, and 68°C for 40 s; and a final extension at 68°C for 4 min. The amplicons were self-ligated using the NEBuilder HiFi DNA Assembly Cloning Kit (New England Biolabs, Inc., Ipswich, MA, USA), as described in the user manual.

### Yeast two-hybrid assay (Y2H)

For expression of each BD-fused phyllogen homolog, the genes were PCR-amplified with the appropriate primers (listed in **Supplementary Table 2**) and cloned into pGBKT7. For expression of the GAL4 activation domain (AD)-fused SVP, *SVP* was cloned into pGADT7 (Clontech, Mountain View, CA, USA). The cloning was performed as described previously (Kitazawa et al., 2017).

To identify phyllogen mutants with enhanced affinity to MTF, the mutant libraries in pGBKT7 were transformed into the *Saccharomyces cerevisiae* Y2H Gold strain (Takara Bio) with a pGADT7-containing AD-fused MTF (*SEP3* or *SVP*). The transformation was performed by the lithium acetate method, as described in the Yeast Protocols Handbook (Clontech). The co-transformants were plated on a synthetically defined (SD) medium lacking leucine, tryptophan, and histidine with 5 mM 3-amino-1,2,4-triazole and 40 mg/L of X-α-Gal (SD/–LWH+3AT+X). After incubation for 4 days at 30°C, colonies were isolated on the same selective medium. The pGBKT7 plasmid was extracted from each isolate using Zymolyase-100T (Nakarai Tesque, Kyoto, Japan) and purified through an antibiotic selection of *Escherichia coli* transformants. The sequences of mutants in the pGBKT7 were determined by Sanger sequencing using the BD_R primer. A second Y2H assay using the purified plasmids and yeast strain AH109 was then performed, as described previously (Kitazawa et al., 2017). Successful co-transformants were selected on SD lacking tryptophan and leucine (SD/–LW) and cultured on three selective media to evaluate the protein–protein interaction: leucine/tryptophan/histidine-lacking SD (SD/–LWH), SD/–LWH containing 5 mM 3-amino-1,2,4-triazole (SD/–LWH+3AT), and leucine/tryptophan/adenine/histidine-lacking SD (SD/–LWAH). The plates were incubated for 4 days at 30°C.

### *In planta* phyllogen expression using a virus vector

A tobacco rattle virus (TRV)-based gene expression vector system (Iwabuchi et al., 2019) was used for systemic gene expression. The *PHYL*_SY_ mutants obtained in the Y2H assay $\left({\mathrm{PHYL}}_{\mathrm{SY}}^{Q30R},\;{\mathrm{PHYL}}_{\mathrm{SY}}^{K37E},\;{\mathrm{PHYL}}_{\mathrm{SY}}^{R64G}\right)$ were cloned into pTRV2 as described previously (Iwabuchi et al., 2019). For expression of $\text{PHY}{\text{L}}_{\text{SY}}^{\text{Q}30\text{R}/\text{R}64\text{G}}$ , *PHYL*_SY_ and ${\mathrm{PHYL}}_{\mathrm{SY}}^{R64G}$ were amplified with the primer pairs 2A-fragariae-F and SYopt_Q30R_R, and SYopt_Q30R_F and pTRV2-fragariae-R, respectively. The amplified fragments were fused by recombinant PCR using KOD FX Neo (Toyobo). The first PCR was performed without primers under the following conditions: 96°C for 3 min; 10 cycles at 96°C for 15 s, 50°C for 30 s, and 68°C for 30 s; and a final extension at 68°C for 4 min. The second PCR was performed with the primer pair of 2A-fragariae-F and pTRV2-fragariae-R, under the same conditions but with 30 cycles of PCR reaction. The fused fragment was cloned into pTRV2 as described above. For cloning of $PHYL_{SY}^{K37E/R64G}$ , DNA fragments were amplified from *PHYL*_SY_ and ${\mathrm{PHYL}}_{\mathrm{SY}}^{R64G}$ with the primer pairs 2A-fragariae-F and SYopt_K37E_R, and SYopt_K37E_F and pTRV2-fragariae-R, respectively; they were then fused and cloned, as in the case of ${\mathrm{PHYL}}_{\mathrm{SY}}^{Q30R/R64G}$ . For expression of $\text{PHY}{\text{L}}_{\text{OY}}^{\text{K}37\text{E}}$ , mutagenesis of TRV2-cloned *PHYL_OY_* (Iwabuchi et al., 2019) was performed using the GeneArt™ Site-Directed Mutagenesis System and the primer pair, OYopt_K37E_F/R (**Supplementary Table 2**).

The virus vectors were inoculated to *A. thaliana* by agroinfiltration with the viral silencing suppressor P19, as described previously (Iwabuchi et al., 2020). The final concentration of each *Agrobacterium* was adjusted to an OD_600_ of 0.1.

### Immunoprecipitation and immunoblotting in *N. benthamiana*

For transient expression in *N. benthamiana* leaves, phyllogens and their mutants, which were created in this study, were PCR-amplified with appropriate primer pairs (**Supplementary Table 2**), cloned into pENTA, and subcloned into pEarleyGateN3 × FLAG (Iwabuchi et al., 2019), as described previously (Kitazawa et al., 2017). *SVP* was also cloned into pEarleyGateC3myc (Okano et al., 2014).

For *in planta* anti-FLAG immunoprecipitation, *Agrobacterium* cultures for the expression of 3 × myc-fused SEP3 and SVP (SEP3- and SVP-3myc, respectively), 3 × FLAG-fused phyllogens (3FLAG-PHYL_SY_, -PHYL_OY_, and -PHYL_WBDL_, or their mutants), and P19, were adjusted to an OD_600_ of 1.0, mixed at a ratio of 1:1:0.1, and infiltrated into *N. benthamiana* leaves. 3 × FLAG-fused phyllogen was immunoprecipitated using EZview Red ANTI-FLAG M2 Affinity Gel (Sigma-Aldrich, St. Louis, MO, USA) 36 h after infiltration, as described previously (Kitazawa et al., 2022). The proteins were separated by SDS-PAGE and detected by immunoblotting, as described previously (Maejima et al., 2014). The commercial antibodies used for immunoblotting included an anti-FLAG antibody (F1804; Sigma-Aldrich) and an anti-myc antibody (4A6; Merck Millipore, Burlington, MA, USA).

For examining SEP3-degradation activity of PHYL_SY_ mutants, *Agrobacterium* cultures for the expression of myc-fused SEP3 (myc-SEP3; Iwabuchi et al., 2019), 3FLAG-PHYL_SY_ or its mutants, and P19, were adjusted to an OD_600_ of 1.0, mixed at a ratio of 1:0.1:0.1, and infiltrated into *N. benthamiana* leaves. Protein extraction and detection were performed according to the previous report (Kitazawa et al., 2022).

### Residue mapping onto the phyllogen structure model

The residues identified to enhance the affinity of phyllogen to MTF were mapped onto the phyllogen protein structure model along with the previously studied residues. The structure model of PHYL_OY_ (PDB ID: 6JQA; Iwabuchi et al., 2019) was used in the study. To map the residues of other phyllogens (e.g., PHYL_SY_), corresponding residues in PHYL_OY_ were selected through alignment of the amino acid sequences of phyllogens using the ClustalW algorithm. Graphic visualization of the protein structure was provided by UCSF Chimera v. 1.9 (https://www.cgl.ucsf.edu/chimera/).

### *In silico* structure prediction of protein complexes

Structures of protein complexes composed of PHYL_OY_ and the K domain of SEP3 (residues 93–145) were predicted by ColabFold using MMseqs2 v. 1.4 (Mirdita et al., 2022). The parameter settings were as follows: use_amber = false, template_mode = none, msa_mode = MMseqs2 (UniRef + Environmental), pair_mode = unpaired + paired, model_type = AlphaFold2-multimer-v2, num_recycles = 6. Mapping of focused residues in the protein complexes was performed using UCSF Chimera v. 1.9.

## Results

### Screening of PHYL_SY_ mutants with enhanced binding affinity to SEP3

**Figure 1:**
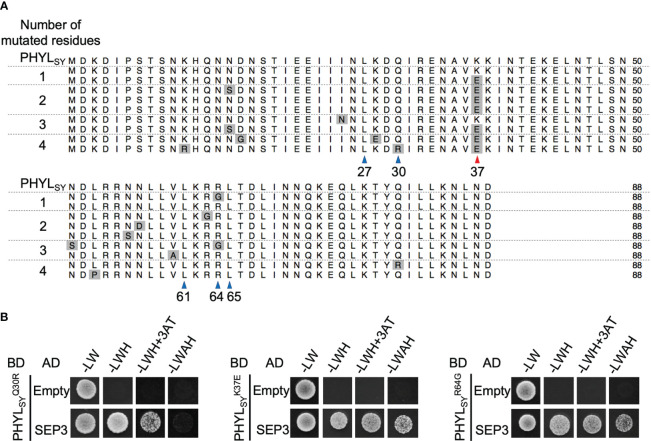
Screening of SEP3-binding PHYL_SY_ mutants. **(A)** Alignment of the protein sequences of PHYL_SY_ and the screened mutants excluding putative signal peptides. Sequences were aligned using the MUSCLE algorithm and grouped by the number of identified amino acid mutations. Mutations are highlighted in gray. Blue and red arrowheads indicate known and newly identified residues involved in the interaction with SEP3, respectively. Numbers under arrowheads denote the positions of amino acid residues on PHYL_SY_ excluding signal peptides. **(B)** Yeast two-hybrid assay using SEP3 and PHYL_SY_ mutants. Yeast cells expressing the GAL4 activation domain (AD)-fused SEP3 and GAL4 binding domain (BD)-fused $\text{PHY}{\text{L}}_{\text{SY}}^{\text{Q}30\text{R}}$ , $\text{PHY}{\text{L}}_{\text{SY}}^{\text{K}37\text{E}}$ , and $\text{PHY}{\text{L}}_{\text{SY}}^{\text{R}64\text{G}}$ were adjusted to an OD_600_ of 0.1. Aliquots (10 μl) of these cells were spotted and incubated on synthetically defined medium lacking leucine/tryptophan (–LW), medium lacking leucine/tryptophan/histidine (–LWH), medium lacking leucine/tryptophan/histidine but containing 5 mM 3-amino-1,2,4-triazole (–LWH+3AT), or medium lacking tryptophan/leucine/adenine/histidine (–LWAH). The assay using SEP3 and PHYL_SY_ was already performed in the same conditions (Iwabuchi et al., 2020), resulting in yeast growth on –LWH but not on –LWH+3AT nor –LWAH (**Supplementary Table 4**).

To identify residues involved in the interaction of phyllogen with MTF, we first created a random mutagenesis library of PHYL_SY_, a non-phyllody-inducing phyllogen with weak affinity to MTFs (Iwabuchi et al., 2020), by error-prone PCR. We performed a Y2H screening of the library to select clones with enhanced binding affinity to SEP3, one of the target MTFs for phyllody-inducing phyllogens, using SD/–LWH+3AT+X media (in which yeast expressing AD-SEP3 and wild-type BD-PHYL_SY_ does not grow). We used AD-SEP3 and BD-phyllogens (or mutants) for the Y2H screening, according to the previous reports (Maejima et al., 2014; Iwabuchi et al., 2020). We obtained nine yeast (Y2H Gold strain) colonies expressing AD-SEP3 and BD-PHYL_SY_ mutants on the media. Amino acid sequence alignment of the PHYL_SY_ mutants from the nine colonies showed that only two clones had an amino acid mutation at position 64 (R64G; from arginine to glycine) (**Figure 1A**), which is a residue previously reported to be responsible for the loss-of-function phenotype of PHYL_SY_ (Iwabuchi et al., 2020). Interestingly, all the other seven clones had an amino acid mutation at position 37 (K37E; from lysine to glutamic acid), as indicated by a red arrowhead. Some mutants had only R64G or K37E mutation. No mutations were found in the other residues known to be involved in the interaction with MTF (Liao et al., 2019; Aurin et al., 2020), except for one clone that had K37E and an additional mutation at position 30 (Q30R; from glutamine to arginine), which is marginally involved in the loss-of-function phenotype of PHYL_SY_ (Iwabuchi et al., 2020). These results suggest that the three mutations can be related to the enhanced binding affinity to SEP3. Then, each mutation was separately introduced into PHYL_SY_ ( $\text{PHY}{\text{L}}_{\text{SY}}^{\text{Q}30\text{R}}$ , $\text{PHY}{\text{L}}_{\text{SY}}^{\text{K}37\text{E}}$ , and $\text{PHY}{\text{L}}_{\text{SY}}^{\text{R}64\text{G}}$ ), and the mutants were subjected to another Y2H assay using AH109 strain as the same conditions as previous reports (**Figure 1B**; **Supplementary Table 4**). The results showed that each mutation enhanced the interaction of PHYL_SY_ with SEP3. Especially, $\text{PHY}{\text{L}}_{\text{SY}}^{\text{K}37\text{E}}$ and $\text{PHY}{\text{L}}_{\text{SY}}^{\text{R}64\text{G}}$ interacted with SEP3 as strongly as PHYL_OY_, a phyllody-inducing phyllogen (**Supplementary Table 4**).

### *In planta* activity of the SEP3-binding PHYL_SY_ mutants

**Figure 2:**
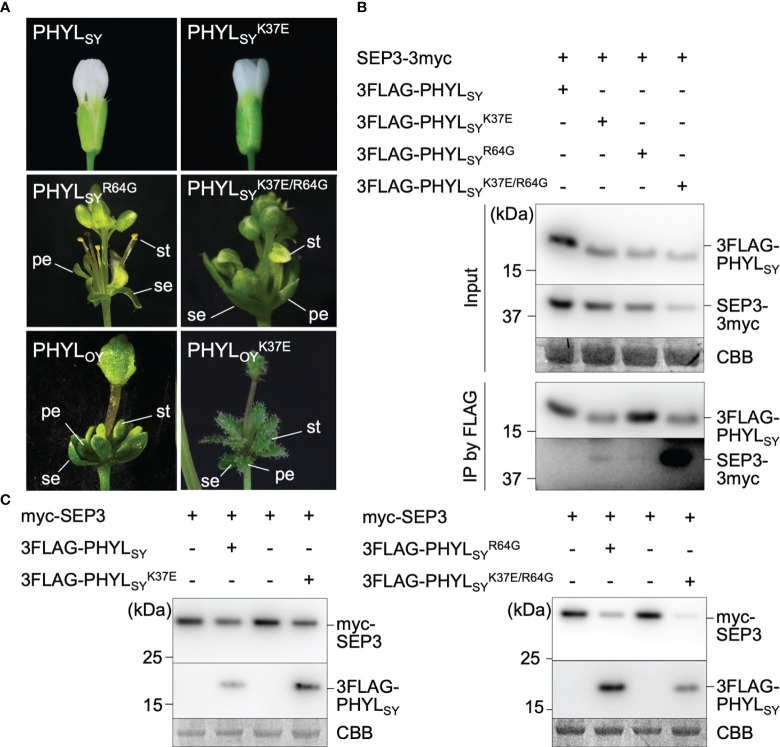
Effects of K37E mutation on phyllogen function. **(A)** Floral phenotypes of *Arabidopsis thaliana* plants infected with the tobacco rattle virus (TRV) vector carrying phyllogens or their mutants. Sepals, petals, and stamens are indicated by (se), (pe), and (st), respectively. **(B)** Interactions of PHYL_SY_ mutants with SEP3 *in planta*. *Agrobacterium* cultures expressing 3×myc tag-fused SEP3 (SEP3-3myc) and either 3×FLAG tag-fused PHYL_SY_ (3FLAG-PHYL_SY_) or its mutants, and P19 were mixed at a ratio of 1:1:0.1 and infiltrated into *Nicotiana benthamiana* leaves. Total proteins were extracted 36 h after infiltration, and immunoprecipitation was performed using an anti-FLAG antibody. The input and immunoprecipitated proteins (IP) were subjected to immunoblot analyses using anti-FLAG and anti-myc antibodies. Coomassie brilliant blue-stained membranes are shown as a loading control (CBB). **(C)** *In planta* accumulation of myc-fused SEP3 (myc-SEP3) in the presence of PHYL_SY_ mutants. *Agrobacterium* cultures (OD = 1.0) for the expression of myc-SEP3, either PHYL_SY_ or its mutants, and P19 were mixed at a ratio of 1:0.1:0.1 and infiltrated into *N. benthamiana* leaves. Protein extraction was performed 36 h after infiltration. Immunoblotting was performed as described above.

The previous report showed that mutation in PHYL_SY_, which enhances its interaction with SEP3, can enhance the interaction with all *Arabidopsis* E-class MTFs (SEP1–4), resulting in the degradation of the MTFs and induction of phyllody in plants (Iwabuchi et al., 2020). Thus, the phyllody-inducing activity in *A. thaliana* was examined for several variants of PHYL_SY_ with mutation(s) that enhance the interaction with SEP3 $\left(\text{PHY}{\text{L}}_{\text{SY}}^{\text{Q}30\text{R}},\text{ PHY}{\text{L}}_{\text{SY}}^{\text{K}37\text{E}},\text{ PHY}{\text{L}}_{\text{SY}}^{\text{R}64\text{G}},\text{ PHY}{\text{L}}_{\text{SY}}^{\text{Q}30\text{R}/\text{R}64\text{G}},\text{ and PHY}{\text{L}}_{\text{SY}}^{\text{K}37\text{E}/\text{R}64\text{G}}\right)$ using the TRV vector. The results showed that $\text{PHY}{\text{L}}_{\text{SY}}^{\text{Q}30\text{R}}$ induced no flower malformation, whereas $\text{PHY}{\text{L}}_{\text{SY}}^{\text{R}64\text{G}}$ induced slight virescence of petals and conversion of a pistil into a stem-like structure (**Supplementary Figure 2**; **Figure 2A**, middle left). $\text{PHY}{\text{L}}_{\text{SY}}^{\text{Q}30\text{R}/\text{R}64\text{G}}$ induced more severe malformations than $\text{PHY}{\text{L}}_{\text{SY}}^{\text{R}64\text{G}}$ , including greenish sterile stamens and strong virescence of petals (**Supplementary Figure 2**). The result indicated that the introduction of Q30R mutation into $\text{PHY}{\text{L}}_{\text{SY}}^{\text{R}64\text{G}}$ strengthens its phyllody-inducing activity. Introduction of K37E mutation showed similar effects to Q30R. Expression of $\text{PHY}{\text{L}}_{\text{SY}}^{\text{K}37\text{E}}$ induced no flower malformation in *A. thaliana* (**Figure 2A**, top right), but the introduction of K37E into $\text{PHY}{\text{L}}_{\text{SY}}^{\text{R}64\text{G}}(\text{PHY}{\text{L}}_{\text{SY}}^{\text{K}37\text{E}/\text{R}64\text{G}})$ enhanced its phyllody-inducing activity, i.e., $\text{PHY}{\text{L}}_{\text{SY}}^{\text{K}37\text{E}/\text{R}64\text{G}}$ induced more severe flower malformations than $\text{PHY}{\text{L}}_{\text{SY}}^{\text{R}64\text{G}}$ (**Figure 2A**, middle lane), such as sterility of stamens and strong virescence of petals. Interestingly, the introduction of K37E into the phyllody-inducing phyllogen PHYL_OY_ further enhanced its phyllody-inducing activity. PHYL_OY_ expression in *A. thaliana* induced green coloration without loss of identity (virescence) of petals and stamens (**Figure 2A**, bottom left); however, $\text{PHY}{\text{L}}_{\text{OY}}^{\text{K}37\text{E}}$ completely transformed petals and stamens into leaf-like structures (**Figure 2A**, bottom right). These results indicated that the introduction of K37E into phyllogens enhances their phyllody-inducing activity.

To investigate the effect of K37E on the *in planta* interaction of PHYL_SY_ with SEP3, FLAG-tag co-immunoprecipitation assays were performed. SEP3-3myc did not co-immunoprecipitate with 3FLAG-PHYL_SY_, as reported previously (Iwabuchi et al., 2020). It weakly co-immunoprecipitated with $3\text{FLAG}-\text{PHY}{\text{L}}_{\text{SY}}^{\text{K}37\text{E}}$ and $3\text{FLAG}-\text{PHY}{\text{L}}_{\text{SY}}^{\text{R}64\text{G}}$ (not detected in some replicates; data not shown), but strongly with $3\text{FLAG}-\text{PHY}{\text{L}}_{\text{SY}}^{\text{K}37\text{E}/\text{R}64\text{G}}$ (**Figure 2B**). Next, the effect of K37E on the *in planta* accumulation of SEP3 was examined. The immunoblotting assay showed that $3\text{FLAG}-\text{PHY}{\text{L}}_{\text{SY}}^{\text{K}37\text{E}}$ did not induce the degradation of myc-SEP3 significantly, as in the case of 3FLAG-PHYL_SY_ (**Figure 2C**). However, $3\text{FLAG}-\text{PHY}{\text{L}}_{\text{SY}}^{\text{K}37\text{E}/\text{R}64\text{G}}$ induced the degradation of myc-SEP3 stronger than $3\text{FLAG}-\text{PHY}{\text{L}}_{\text{SY}}^{\text{R}64\text{G}}$ . These results indicated that K37E enhances the affinity of PHYL_SY_ to SEP3 synergistically with R64G.

### Screening of phyllogen mutants with enhanced binding affinity to SVP

**Figure 3:**
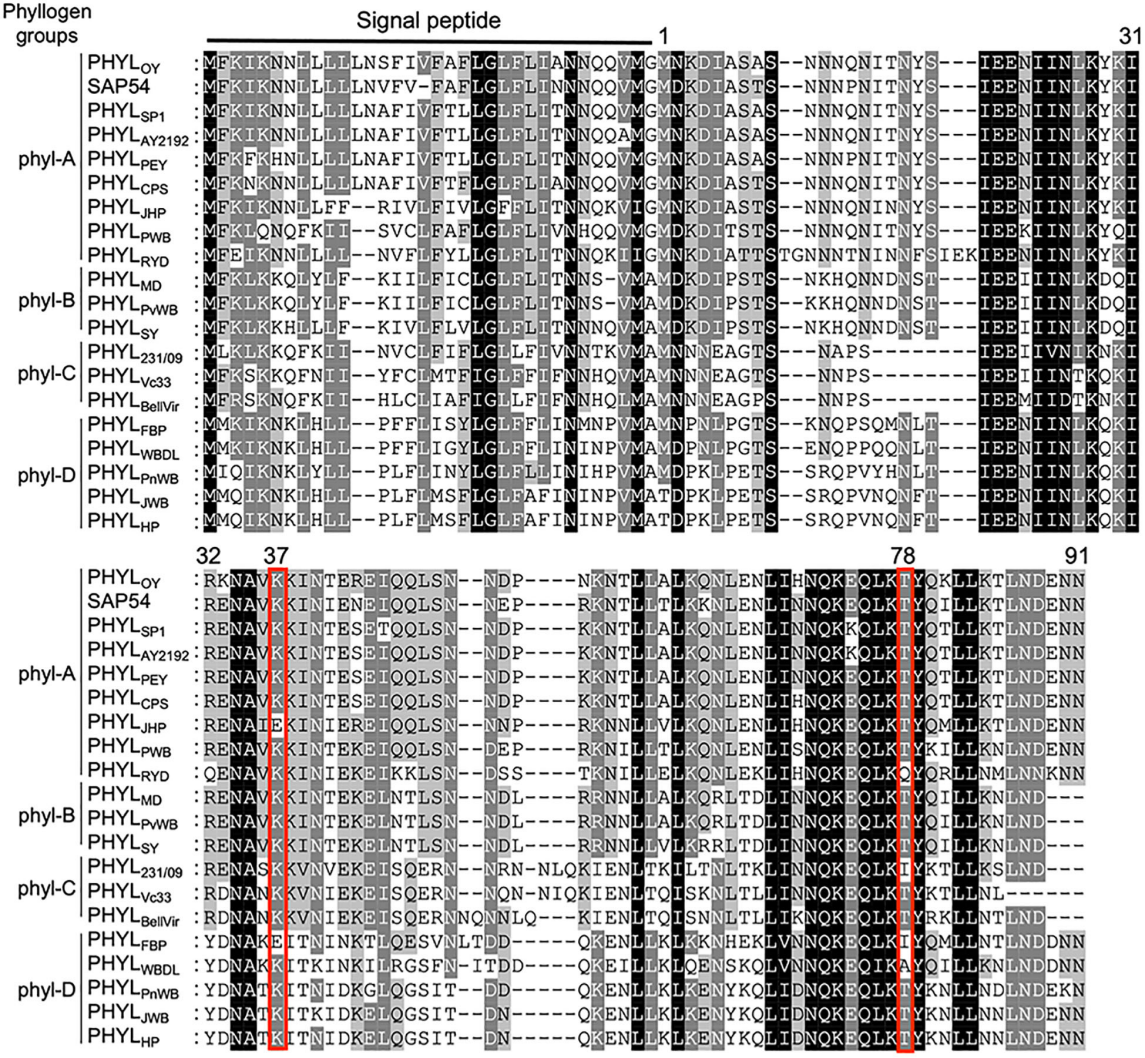
Amino acid polymorphisms in phyllogens. Red lines indicate residues newly identified to be involved in the interaction with MTF. Sequences of phyllogen homologs were retrieved from a previous report (Iwabuchi et al., 2020). Phyllogen groups distinguished by phylogenetic analyses of nucleotide sequences (phyl-A– phyl-D; Iwabuchi et al., 2020) are shown on the left. The background indicates the percentage of amino acid similarity: black, 100%; dark gray, 80%; light gray, 60%. Numbers indicate the positions of amino acid residues of PHYL_OY_ excluding signal peptides.

To further explore the residues of phyllogen related to the interaction with MTFs, we attempted to identify phyllogen mutants with enhanced binding affinity to various MTFs, i.e., AGAMOUS (AG; C-class), APETALA3 (AP3; B-class), and SVP (non-ABCE-class type II MTF) (Smaczniak et al., 2012). These MTFs have not been confirmed to interact with phyllogen *in planta*, although gene expression of AP3 and SVP was shown to be indirectly affected by phyllogen (Maejima et al., 2014; Yang et al., 2015). We performed Y2H screening using mutant libraries of PHYL_OY_ and PHYL_SY_, but only SVP-binding PHYL_OY_ mutants were obtained. All the mutants had the same mutation, i.e., T78A (from threonine to alanine), and PHYL_OY_ with a point mutation $\left(\text{PHY}{\text{L}}_{\text{OY}}^{\text{T}78\text{A}}\right)$ interacted more strongly with SVP than PHYL_OY_ in yeast (**Table 1** and **Supplementary Figure 3**). Sequence alignment analysis showed that the residue T78A is polymorphic among phyllogen homologs (**Figure 3**, Iwabuchi et al., 2020); therefore, Y2H was performed using SVP and the phyllogen homologs with representative amino acid polymorphisms at the corresponding residue (**Table 1**; **Supplementary Figure 3B**). The T78-type phyllogens [SAP54 (a phyllogen homolog from AY-WB phytoplasma), PHYL_SY_, and PHYL_PnWB_], except for PHYL_JHP_, showed no or weak interaction with SVP, as in the case of PHYL_OY_. On the other hand, the non-T78-type phyllogens (PHYL_RYD_, PHYL_231/09_, and PHYL_WBDL_) interacted strongly with SVP. When PHYL_WBDL_ was mutated to a T78-type phyllogen $\left(\text{PHY}{\text{L}}_{\text{WBDL}}^{\text{A}79\text{T}}\right)$ , the interaction with SVP was weakened. These results indicated that the amino acid polymorphisms at this position affect the interaction between phyllogen and SVP in yeast.

**Table 1:** Yeast two-hybrid assay using SVP and phyllogens.

Phyllogen group^a^	Phyllogen	Polymorphism at position 78 of PHYL_OY_	Growth of yeast
–	empty	–	–
phyl-A	PHYL_OY_	T (78)^b^	+
	$\text{PHY}{\text{L}}_{\text{OY}}^{\text{T}78\text{A}}$	A (78)	++
	PHYL_JHP_	T (78)	++
	SAP54	T (78)	–
	PHYL_RYD_	Q (83)	++
phyl-B	PHYL_SY_	T (78)	–
phyl-C	PHYL_231/09_	I (75)	++
phyl-D	PHYL_WBDL_	A (79)	++
	$\text{PHY}{\text{L}}_{\text{WBDL}}^{\text{A}79\text{T}}$	T (79)	+
	PHYL_PnWB_	T (77)	+

++the yeast grew on −LWH+3AT, −LWH, and −LW; +: the yeast grew on −LWH and −LW; -: the yeast grew on only −LW.

a Grouping is based on phylogenetic analysis using nucleotide sequences (Iwabuchi et al., 2020).

b Numbers in parentheses show the amino acid position of the residue in each phyllogen homolog.

Then, the *in planta* interaction of $\text{PHY}{\text{L}}_{\text{OY}}^{\text{T}78\text{A}}$ and SVP was examined by co-immunoprecipitation assay (**Supplementary Figure 4**). The results showed that SVP-3myc did not co-immunoprecipitate with $3\text{FLAG}-\text{PHY}{\text{L}}_{\text{OY}}^{\text{T}78\text{A}}$ . Furthermore, *in planta* interaction of SVP-3myc and 3FLAG-PHYL_WBDL_ was not detected. These results indicated that the mutation T78A is insufficient to enhance the interaction of phyllogen with SVP *in planta* at a detectable level.

### Mapping of residues related to interactions with MTFs onto the phyllogen structure model

**Figure 4:**
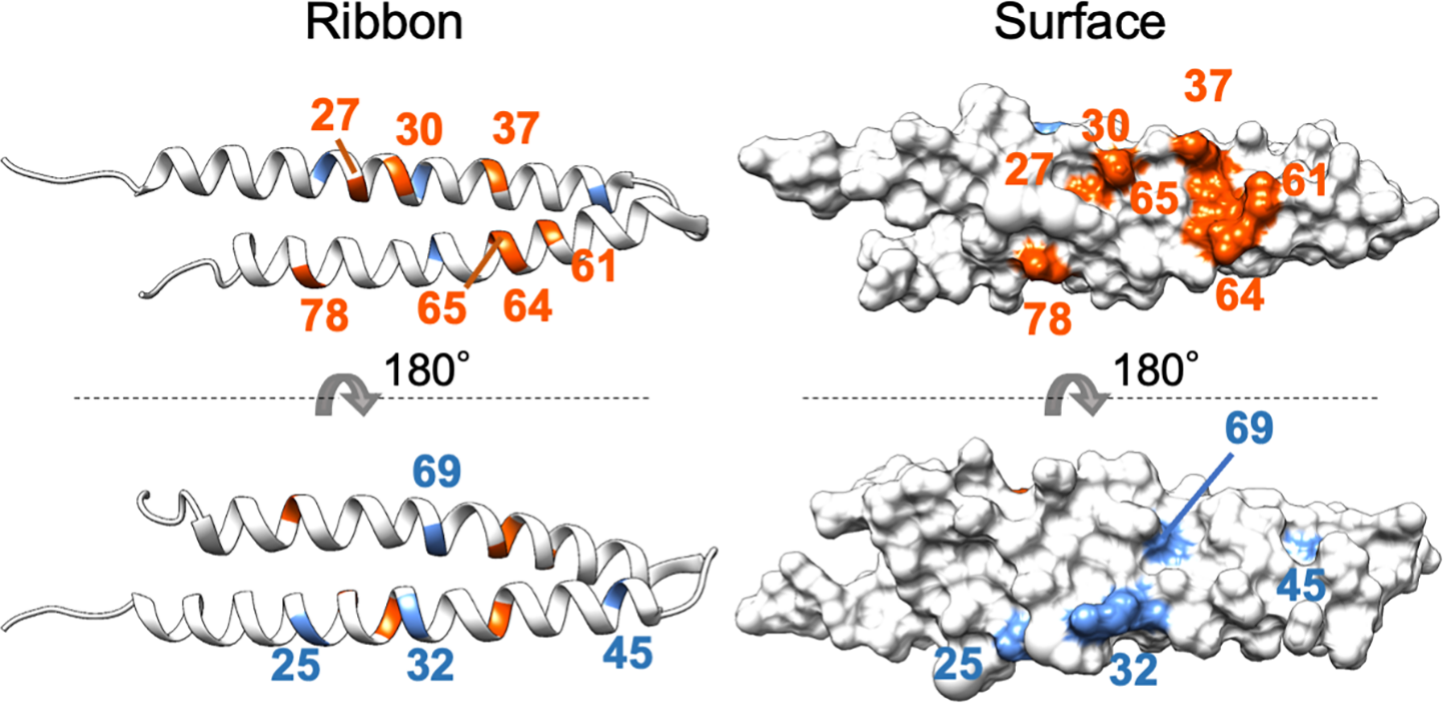
Positions of residues affecting interactions with MTFs on phyllogen. The ribbon and surface structure of PHYL_OY_ (PDB ID: 6JQA) is shown. The colored residues represent those whose mutations were shown to affect (orange) or not affect (blue) interaction with MTFs, as shown in **Supplementary Table 5**. Numbers represent the positions of amino acid residues in PHYL_OY_. For residues in other phyllogens, the corresponding residues in PHYL_OY_ are shown.

The mutagenesis analysis showed that K37E of PHYL_SY_ and T78A of PHYL_OY_ enhanced their interactions with MTFs (**Figure 1** and **Supplementary Figure 3**), suggesting that these amino acid residues constitute, or are at least near, the interface at which phyllogens interact with MTFs. To characterize the interface, these residues as well as the other residues known to be related to interactions with MTFs (**Supplementary Table 5**) were mapped onto the PHYL_OY_ structure model (**Figure 4**). We used PHYL_OY_ because its *in planta* interaction with SEP3 was confirmed (Kitazawa et al., 2022) and its protein structure was determined (Iwabuchi et al., 2019). For mapping of the interaction-related residues in other phyllogens (e.g., PHYL_SY_), corresponding residues in PHYL_OY_ were selected through the alignment of the amino acid sequences of the phyllogens. The residues, at which mutations enhanced the affinity of phyllogen to MTF, identified in this study (positions 37, 78) and the previous studies (positions 27, 30, 61, 64, 65) were shown in orange. Interestingly, the residues were exposed on the same side of the PHYL_OY_ protein surface. On the other hand, the previously reported residues whose mutations do not affect interactions with MTFs (positions 25, 32, 45, 69; blue) were exposed on the opposite side of the protein.

**Figure 5:**
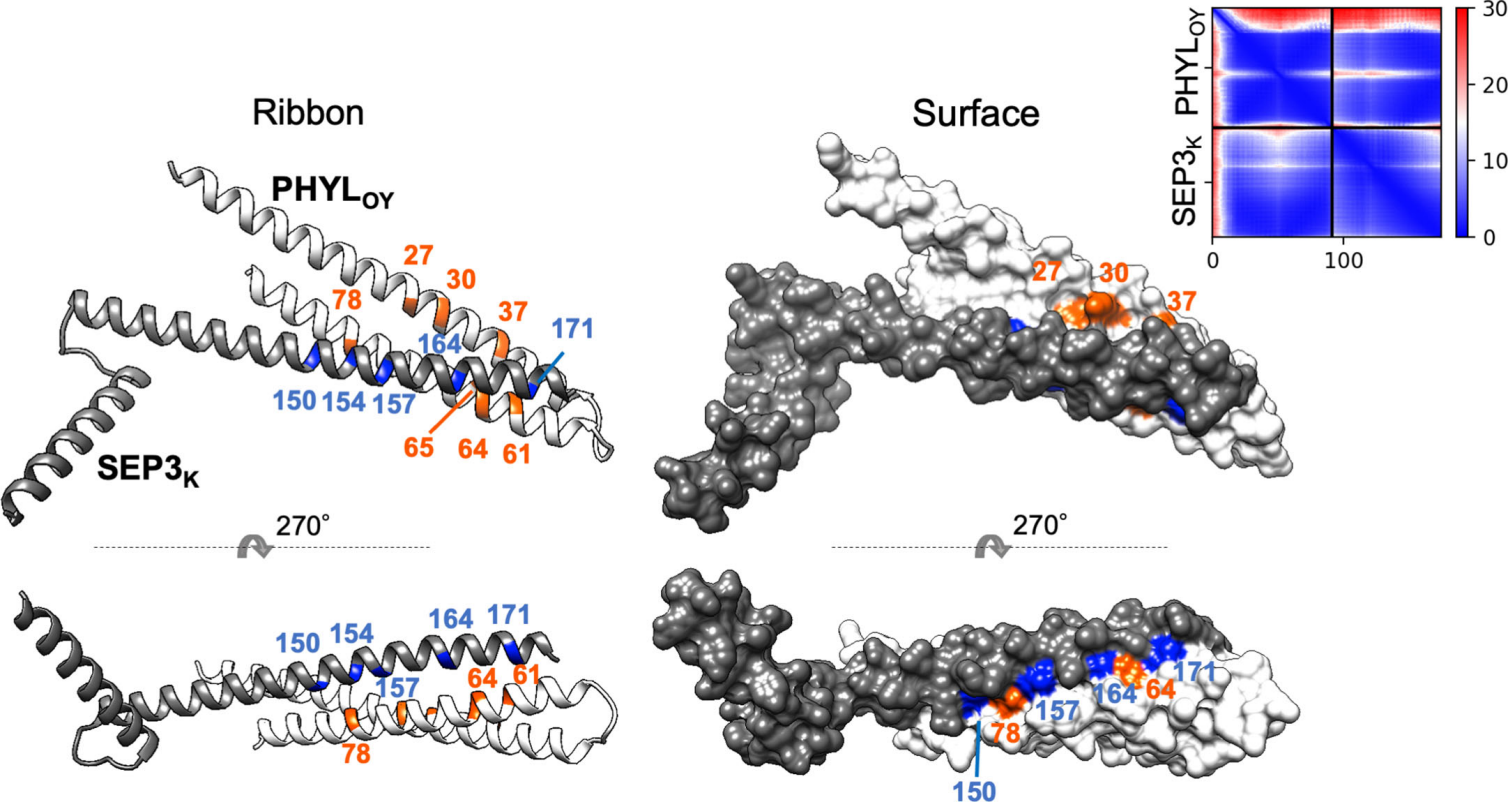
*In silico* structure prediction of the protein complex consisting of PHYL_OY_ and the K domain of SEP3. The structure was predicted by ColabFold. The predicted ribbon and surface structure of PHYL_OY_ and the K domain of SEP3 (SEP3_K_) are shown in white and gray, respectively. The orange-colored residues in PHYL_OY_ represent those whose mutations were shown to affect interaction with MTFs. The blue-colored residues in SEP3_K_ represent the tetramerization-related residues (Puranik et al., 2014). Numbers represent the positions of amino acid residues in each protein. The predicted aligned error (PAE) for each residue pair was calculated by ColabFold and is shown in the upper right corner.

Finally, a model of a protein complex composed of PHYL_OY_ and the K domain of SEP3 (SEP3_K_) was constructed *in silico* (**Figure 5**) using ColabFold (Mirdita et al., 2022). The predicted local distance difference test (pLDDT), predicted TM (pTM), and interface pTM (ipTM) scores used for estimating model accuracy were 91.5, 0.787, and 0.792, respectively. These scores are above or near the thresholds proposed for high confidence prediction (85, 0.8, and 0.75, respectively) (Mirdita et al., 2022; Yin et al., 2022). In the predicted model, PHYL_OY_ interacts with the tetramerization-involved region in SEP3_K_, as shown experimentally in our previous report (Kitazawa et al., 2022). Moreover, the surface of PHYL_OY_ on which the interaction-involved residues were exposed was near the surface of SEP3_K_ constituted by the five tetramerization-involved residues (Puranik et al., 2014), which is a putative interface for the interaction with phyllogen (Kitazawa et al., 2022). Especially, N64 of PHYL_OY_ was predicted to be located near two tetramerization-involved SEP3_K_ residues (Leu164 and Leu171).

## Discussion

Random mutagenesis can be performed without *a priori* prediction of the effect of mutations. Thus, this approach is useful for obtaining mutations enhancing or decreasing protein–protein interactions. In this study, random mutagenesis screening of phyllogen revealed two mutations that enhance affinity to MTFs, at amino acid residues previously unknown to be involved in this interaction. Interestingly, the two residues were located on the same side of the protein as the residues previously reported to be involved in the interaction (**Figure 4**). Moreover, the surface of the phyllogen protein constituted by these residues was predicted *in silico* to interact with the surface of SEP3_K_ constituted by the tetramerization-involved residues (**Figure 5**). The predicted model was consistent with a previous study showing that mutations in the five tetramerization-involved residues of SEP3_K_ disrupt its interaction with phyllogen (Kitazawa et al., 2022). The validity of the predicted model should be verified further, and the stoichiometry of the protein complex has not been elucidated. However, taken together, the mutation screening and the *in silico* prediction strongly indicated that the surface of phyllogen, where the interaction-involved residues are exposed, is an interface for the interaction with MTF. Further analyses focusing on residues on this surface will provide further insights into the mode of interaction.

Positions 37 in PHYL_SY_ and 78 in PHYL_OY_ were residues not previously reported to affect interactions with MTFs. Although the mutations were introduced and selected randomly, the same or similar polymorphisms already exist in some phyllogen homologs in various phytoplasmas (**Figure 3**). For example, PHYL_JHP_ and PHYL_FBP_ have E37, and PHYL_WBDL_ has A79. This suggests that each phyllogen may have evolved to differentiate the binding affinity to targets and modulate target specificity by obtaining polymorphisms in interaction-involved residues. The Y2H assay showed that non-T78-type phyllogens, which have an amino acid polymorphism at the residue corresponding to T78 of PHYL_OY_, interact more strongly with SVP in comparison to T78-type phyllogens (**Table 1**). Although it is reported that SAP54, a phyllogen from AY-WB phytoplasma, interacts with a wide variety of MTFs (MacLean et al., 2014), the interactions between other phyllogen homologs and MTFs other than A- and E-class MTFs have not been elucidated. If each homolog targets different MTFs in addition to the consensus A- and E-class targets, it might have a different effect on plants. Thus, exploring the target specificity and binding affinity of each phyllogen homolog should promote a comprehensive understanding of the functions of the phyllogen family.

We previously showed that a mutation in PHYL_SY_ based on natural genetic polymorphisms (Q30K or R64N) enhances its binding affinity to MTF and its phyllody-inducing activity (marginally or markedly, respectively) (Iwabuchi et al., 2020). Our random mutagenesis screening also showed that the exact same residues, albeit with different mutations (Q30R and R64G), enhance the binding affinity and/or phyllody-inducing activity (**Supplementary Table 4**, **Supplementary Figure 2**). We could not obtain $\text{PHY}{\text{L}}_{\text{SY}}^{\text{R}64\text{N}}$ or $\text{PHY}{\text{L}}_{\text{SY}}^{\text{Q}30\text{K}}$ in the screening, which may be due to biases in amino acid mutations introduced by error-prone PCR (Neylon, 2004), such as codon bias [e.g., R64N requires two nucleotide substitutions (AGG to AAC or ATT)], and the tendency for nucleotide substitution (e.g., A to G and T to C are the most frequent substitutions according to the user manual of the Diversify PCR Random Mutagenesis Kit). This may also explain why we failed to obtain phyllogen mutants interacting with AG and AP3. Therefore, other interaction-involved residues may be unidentified. Reducing these biases by modifying the experimental conditions and codon usage of the phyllogen genes as a PCR template will help us obtain novel phyllogen mutants with enhanced binding affinity to MTFs of interest. Because MTFs regulate diverse pathways in plant development (Smaczniak et al., 2012), this approach could lead to the use of phyllogen as a tool for regulating MTF functions and modulating plant architecture.

## Data availability statement

The original contributions presented in the study are included in the article/**Supplementary Material**. Further inquiries can be directed to the corresponding author.

## Author contributions

YK, NI, and KM designed the research. HK, KO, SN, and YY supervised the experiments. YK performed most experiments. OM, MS, and JM provided technical assistance in the experiments. YK wrote the initial draft of the article. OM, MS, and JM complemented the writing. NI, KM, HK, KO, SN, and YY critically revised and edited the manuscript. All authors contributed to the article and approved the submitted version.
