# Supplementary material for: Random mutagenesis-based screening of the interface of phyllogen, a bacterial phyllody-inducing effector, for interaction with plant MADS-box proteins
Source: Front Plant Sci. 2023 Mar 28;14:1058059. doi: 10.3389/fpls.2023.1058059 (PMC10086140; doi:10.3389/fpls.2023.1058059)
Supplement: Supplementary file 1 [file DataSheet_1.docx]

>PHYL_JHP__opt

ATGAACAAGGATATCGCTTCTACCTCTAACAACAACCAGAACATCAACAACTACTCTATCGAAGAGAACATTATCAACCTCAAGTACAAGATCAGAGAGAACGCTATCGAGAAGATCAACATCGAGAGAGAGATCCAGCAGCTCTCAAACAACAATCCTAGGAAGAACAACCTCCTCGTTCTCAAGCAGAACCTCGAGAACCTCATCCATAACCAGAAAGAGCAGCTCAAGACCTACCAGATGCTCCTCAAGACTCTCAACGATGAGAACAACTGA

>PHYL_RYD__opt

ATGAACAAGGATATCGCTACCACCTCTACCGGAAACAACAACACCAACATCAACAACTTCTCTATCGAGAAGATCGAAGAGAACATCATCAACCTCAAGTACAAGATTCAAGAGAACGCTGTGAAGAAGATCAACATCGAGAAAGAGATCAAGAAGCTCTCTAACGATTCTTCTACCAAGAACATCCTCCTCGAGCTTAAGCAGAACCTCGAGAAGCTCATCCACAACCAGAAAGAGCAGCTCAAGCAGTACCAGAGGCTTCTCAACATGCTCAACAACAAGAACAACTGA

>PHYL_WDBL__opt

ATGGATCCTAACCTCCCTGGAACCTCTGAGAATCAACCTCCTCAGCAGAACCTCACCATCGAAGAGAACATCATCAACCTCAAGCAAAAGATCTACGATAACGCTAAGAAGATCACCAAGATTAACAAGATCCTCAGGGGATCTTTCAACATCACCGATGATCAGAAAGAGATCCTCCTCAAGCTCCAAGAGAACTCTAAGCAGCTCGTGAACAACCAGAAAGAGCTTATCAAGGCTTACCAGATCTTGCTCAAGAACCTCAACGATGATAACAACTGA
