# Supplementary material for: Random mutagenesis-based screening of the interface of phyllogen, a bacterial phyllody-inducing effector, for interaction with plant MADS-box proteins
Source: Front Plant Sci. 2023 Mar 28;14:1058059. doi: 10.3389/fpls.2023.1058059 (PMC10086140; doi:10.3389/fpls.2023.1058059)
Supplement: Supplementary file 2 [file Image_1.pdf]

$PHYL_{JHP}$

original ATGAATAAAGATATTGCTAGCACTAGTAATAATAATCAAAACATCAATAA 50
optimized ATGAACAAGGATATCGCTTCTACCTCTAACAACAACCAGAACATCAACAA 50

original TTACTCTATTGAAGAAAATATAATTAATTTAAAATATAAAATTCGGGAAA 100
optimized CTACTCTATCGAAGAGAACATTATCAACCTCAAGTACAAGATCAGAGAGA 100

original ATGCAATTGAAAAAATAAATATAGAAAGAGAAATACAACAATTATCAAAT 150
optimized ACGCTATCGAGAAGATCAACATCGAGAGAGAGATCCAGCAGCTCTCAAAC 150

original AATAATCCTAGAAAAAATAATCTTTTAGTGTTGAAACAAAATTTAGAAAA 200
optimized AACAATCCTAGGAAGAACAACCTCCTCGTTCTCAAGCAGAACCTCGAGAA 200

original TTTAATTCATAATCAAAAAGAACAATTAAAAACTTATCAAATGCTTTTAA 250
optimized CCTCATCCATAACCAGAAAGAGCAGCTCAAGACCTACCAGATGCTCCTCA 250

original AGACTTTAAATGATGAAAATAATTAA 276
optimized AGACTCTCAACGATGAGAACAACTGA 276

$PHYL_{RYD}$

original ATGAATAAAGATATTGCTACTACTAGCACTGGTAATAATAATACAAACAT 50
optimized ATGAACAAGGATATCGCTACCACCTCTACCGGAAACAACAACACCAACAT 50

original AAATAATTTTTCTATTGAAAAAATTGAAGAAAATATAATTAATTTAAAAT 100
optimized CAACAACTTCTCTATCGAGAAGATCGAAGAGAACATCATCAACCTCAAGT 100

original ATAAAATTCAAGAAAACGCAGTTAAAAAAAATAAATATAGAAAAAGAAATA 150
optimized ACAAGATTCAAGAGAACGCTGTGAAGAAGATCAACATCGAGAAAGAGATC 150

original AAAAAATTATCTAATGATAGTTCTACAAAAAATATTCTTTTAGAATTAAA 200
optimized AAGAAGCTCTCTAACGATTCTTCTACCAAGAACATCCTCCTCGAGCTTAA 200

original ACAAAATTTAGAAAAATTAATTCATAATCAAAAAGAACAATTAAAACAAT 250
optimized GCAGAACCTCGAGAAGCTCATCCACAACCAGAAAGAGCAGCTCAAGCAGT 250

original ATCAAAGACTTTTAAACATGTTAAATAATAAAAATAATTAA 291
optimized ACCAGAGGCTTCTCAACATGCTCAACAACAAGAACAACTGA 291

$PHYL_{WBDL}$

original ATGGATCCAAACCTTCCAGGAACTAGTGAAAATCAACCTCCTCAACAGAA 50
optimized ATGGATCCTAACCTCCCTGGAACCTCTGAGAATCAACCTCCTCAGCAGAA 50

original TCTCACTATTGAAGAAAACATCATTAATTTAAAACAAAAAATTTATGATA 100
optimized CCTCACCATCGAAGAGAACATCATCAACCTCAAGCAAAAGATCTACGATA 100

original ATGCAAAAAAAATAACAAAAATAAATAAAATATTACGAGGATCATTTAAT 150
optimized ACGCTAAGAAGATCACCAAGATTAACAAGATCCTCAGGGGATCTTTCAAC 150

original ATCACTGATGATCAAAAAGAAATTCTCTTAAAATTACAAGAAAATAGCAA 200
optimized ATCACCGATGATCAGAAAGAGATCCTCCTCAAGCTCCAAGAGAACTCTAA 200

original ACAATTAGTTAATAATCAAAAAGAACAAATAAAAGCTTATCAAATTCTTT 250
optimized GCAGCTCGTGAACAACCAGAAAGAGCTTATCAAGGCTTACCAGATCTTGC 250

original TAAAGAATTTAAATGATGACAATAACTAA 279
optimized TCAAGAACCTCAACGATGATAACAACTGA 279

Supplementary Figure 1. Optimized nucleotide sequences of putative secreted regions of phyllogens cloned in the study. Optimized nucleotides were highlighted in gray.

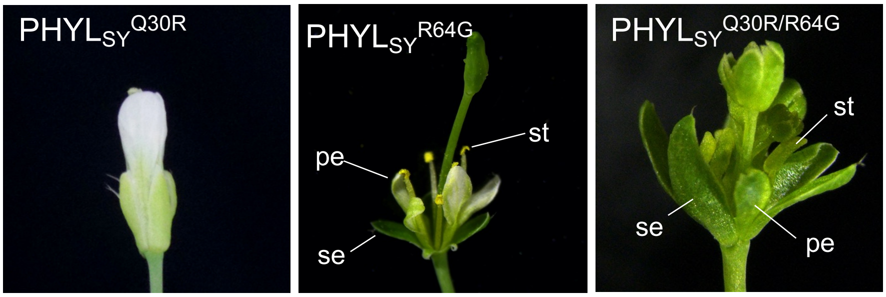

Supplementary Figure 2. Effects of mutation Q30R and/or R64G in PHYL$_{SY}$. Floral phenotypes of *Arabidopsis thaliana* plants infected with the tobacco rattle virus (TRV) vector carrying PHYL$_{SY}$ mutants were shown. Sepals, petals, and stamens are indicated as (se), (pe), and (st), respectively.

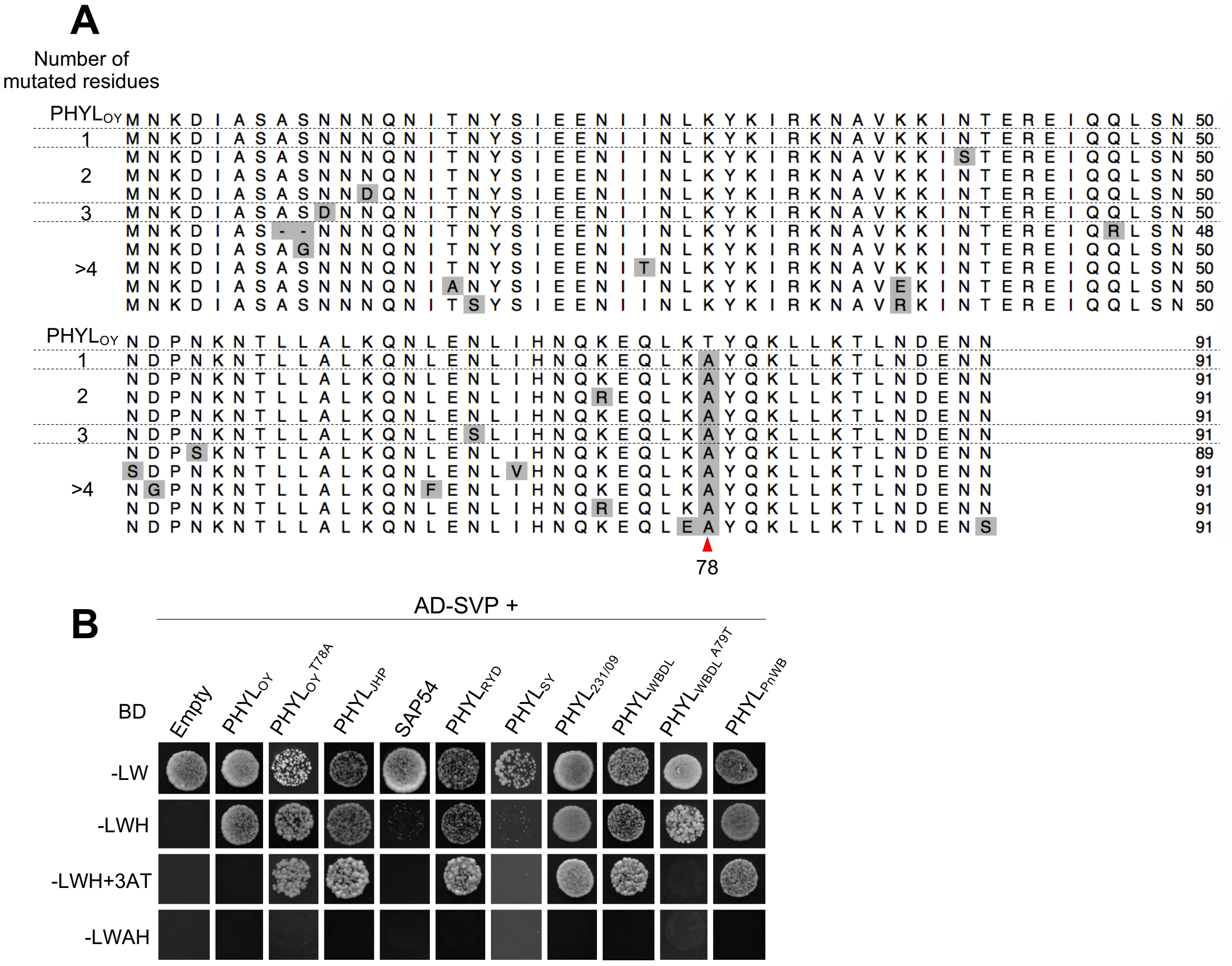

Supplementary Figure 3. Interactions of phyllogens with SVP in yeast. **(A)** Alignment of the protein sequences of $PHYL_{OY}$ and the screened mutants excluding putative signal peptides. Sequences were aligned using the MUSCLE algorithm. Mutations were highlighted in gray. A red arrowhead indicates an interaction-involved mutation T78A, numbered based on amino acid sequences of $PHYL_{OY}$ without signal peptides. **(B)** Yeast two hybrid assay using SVP and $PHYL_{OY}$, $PHYL_{OY}^{T78A}$ and other phyllogen homologs. The experimental conditions were described in **Figure 1B**.

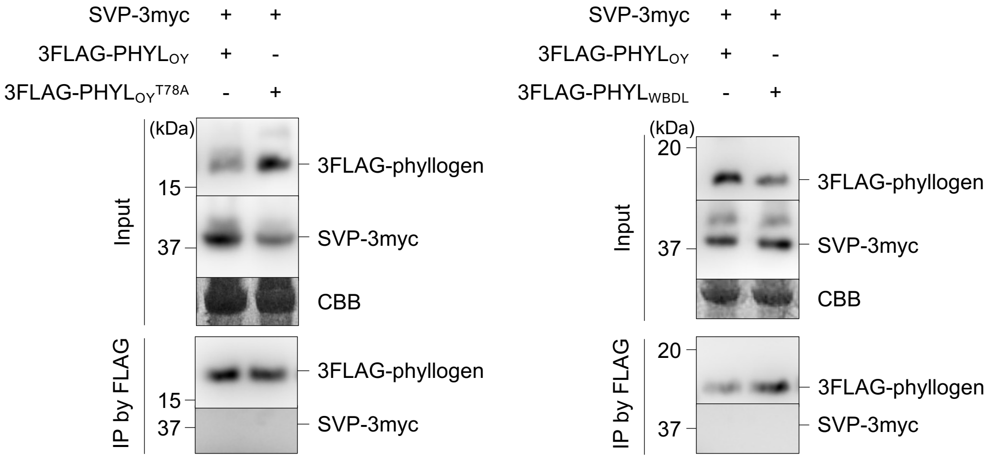

Supplementary Figure 4. Interactions of phyllogens with SVP *in planta* were not detected. Co-immunoprecipitation assays were performed using 3 × myc tag-fused SVP (SVP-3myc) and 3 × FLAG tag-fused PHYL$_{OY}$ (3FLAG-PHYL$_{OY}$) and either of its mutant (Left) or PHYL$_{WBDL}$ (Right). The experimental conditions were described in **Figure 2**.
